# The Impact of Comorbid Anxiety and Depression on qEEG in Adults with Attention-Deficit Hyperactivity Disease

**DOI:** 10.1192/j.eurpsy.2026.11762

**Published:** 2026-07-31

**Authors:** J. Jeong, S. Kim, Y. C. Kim

**Affiliations:** Psychiatry, St. Vincent’s Hospital, The Catholic Universit of Korea, Suwon, Korea, Republic Of

## Abstract

**Introduction:**

Attention-Deficit Hyperactivity Disorder (ADHD) is a neurodevelopmental disorder that frequently persists into adulthood, with a global prevalence of 2.3–4.5%. Diagnosing adult ADHD is challenging due to retrospective symptom recall difficulties and frequent psychiatric comorbidities, particularly depression and anxiety, which share overlapping symptoms.

**Objectives:**

Quantitative electroencephalography (qEEG) has emerged as a potential diagnostic tool by identifying neurophysiological markers, such as frontal alpha asymmetry, that differentiate ADHD from comorbid conditions. This study examines qEEG differences between individuals with ADHD alone and those with comorbid depression and anxiety, hypothesizing distinct neurophysiological patterns

**Methods:**

This retrospective study included 51 young adult outpatients (age: 18–30 years) diagnosed with ADHD, of whom 30 (58.8%) had comorbid anxiety and depression. qEEG was performed on (left, middle, right) and theta/beta ratio (TBR). Frontal medication status, and FSIQ as covariates. both groups, focusing on absolute power in three frontal regions asymmetry was analyzed across prefrontal (Fp2-Fp1), middle frontal (F4-F3), and lateral frontal (F8-F7) regions for all frequency bands. Ranked ANCOVA was used to compare qEEG differences between groups, with age, sex, current.

**Results:**

Demographic characteristics and neuropsychological test scores did not differ significantly between groups. However, significant differences were observed in frontal asymmetry in theta waves in the lateral frontal region (p = 0.03), indicating potential neurophysiological differences between ADHD patients with and without comorbidities. No significant differences were found in other frequency bands or brain regions between the two groups

**Conclusions:**

ADHD patients with comorbid depression and anxiety exhibited reduced frontal asymmetry in the lateral frontal region, particularly in theta waves, compared to those with ADHD alone. This suggests that psychiatric comorbidities may modulate neurophysiological patterns in ADHD, potentially influencing qEEG findings. These results support the utility of qEEG as a potential diagnostic tool for distinguishing ADHD subtypes, particularly in patients with coexisting anxiety and depression. However, further studies with larger sample sizes are necessary to confirm these findings and explore their clinical implications.

**Disclosure of Interest:**

None Declared

